# The present and potential future methods for delivering CRISPR/Cas9 components in plants

**DOI:** 10.1186/s43141-020-00036-8

**Published:** 2020-07-07

**Authors:** Dulam Sandhya, Phanikanth Jogam, Venkateswar Rao Allini, Sadanandam Abbagani, Anshu Alok

**Affiliations:** 1 Department of Biotechnology, Kakatiya University, Warangal, Telangana India; 2 Department of Biotechnology, UIET, Panjab University, Chandigarh, India

**Keywords:** CRISPR/Cas9, Floral-dip, PEG, Pollen magnetofection, Genome editing

## Abstract

**Background:**

CRISPR/Cas9 genome editing technology is a DNA manipulation tool for trait improvement. This technology has been demonstrated and successfully applied to edit the genome in various species of plants. The delivery of CRISPR/Cas9 components within rigid plant cells is very crucial for high editing efficiency. Here, we insight the strengths and weaknesses of each method of delivery.

**Main text:**

The mutation efficiency of genome editing may vary and affected by different factors. Out of various factors, the delivery of CRISPR/Cas9 components into cells and genome is vital. The way of delivery defines whether the edited plant is transgenic or transgene-free. In many countries, the transgenic approach of improvement is a significant limitation in the regulatory approval of genetically modified crops. Gene editing provides an opportunity for generating transgene-free edited genome of the plant. Nevertheless, the mode of delivery of the CRISPR/Cas9 component is of crucial importance for genome modification in plants. Different delivery methods such as *Agrobacterium*-mediated, bombardment or biolistic method, floral-dip, and PEG-mediated protoplast are frequently applied to crops for efficient genome editing.

**Conclusion:**

We have reviewed different delivery methods with prons and cons for genome editing in plants. A novel nanoparticle and pollen magnetofection-mediated delivery systems which would be very useful in the near future. Further, the factors affecting editing efficiency, such as the promoter, transformation method, and selection pressure, are discussed in the present review.

## Background

Plants play a vital role in human life by offering a variety of plant-based products such as fruits, food grains, vegetables, and medicine. The various traits of plants can be improved by plant breeding and genetic engineering activities [1]. Plant genetic engineering can be achieved utilizing multiple tools such as overexpression, RNA interference, Zinc finger TALEN nuclease, and CRISPR/Cas9 [2–5]. CRISPR/Cas9 genome editing tool is derived from the bacterial CRISPR system, which is known to involve in the immune system. CRISPR/Cas9 technology has gained tremendous popularity due to its specificity and efficiency in editing the genome. Several CRISPR/Cas9 and its variants have been applied for the genome editing of many desired genes. The CRISPR (clustered regularly interspaced short palindromic repeats) locus and its associated proteins are basically found in few bacteria, and it is related to immunity against phages. The different regions of CRISPR locus transcribed and lead to the formation of CRISPR RNA (crRNA) and trans-activating CRISPR RNA (tracrRNA). The crRNA, tracrRNA, and Cas9 encounter the phage DNA. The guide RNA (gRNA) is a synthetic gene comprised of crRNA and tracrRNA [6].

The Cas9 gene and gRNA under the regulation of the appropriated promoters within any vector, can be delivered into the plant cells. In another approach, the Cas9, also known as RNA guided site-specific nucleases (RGNs) and transcribed gRNA, is assembled and then delivered into plant regenerative tissue. The target site must contain 5′NGG3′ for the action of the CRISPR/Cas9 system, which is also known as Protospacer Adjacent Motif (PAM). Cas9 cleaves both the strands of a target gene or DNA with the help of gRNA. This double-stranded breaks (DSBs) may be restored by either homologous direct repair (HDR) or non-homologous end joining (NHEJ) via a repair mechanism [7]. During DNA repair, the insertion or deletion of nucleotide results in the point mutation or frameshift mutation. These mutations are generally identified by various techniques; however, the restriction enzyme site loss assay, AFLP, and Sanger-based sequencing are frequently used [8, 9].

Different strategies have been developed to target multiple genes at a time, i.e., multiplexing [10, 11]. This multiplex genome engineering is generally used to target various genes within the genome or distinct target within one gene to increase mutation efficiency. This technique involves the expression of multiple gRNAs under different promoters or single promoter using the polycistronic gRNA unit [11]. Polycistronic gRNA unit is assembled with the help of either tRNA-gRNA or Cys4-gRNA. This single synthetic gene is transcribed by a single promoter. The RNases P and Z enzymes cleave polycistronic tRNA-gRNA. Csy4 (CRISPR system yersinia 4) is an RNA nuclease characterized from *Pseudomonas aeruginosa* separate polycistronic Cys4-gRNA into individual gRNA [11]. The tRNA processing enzymes are naturally present in almost all living organisms, including plant cells [12]. This technology has been demonstrated and applied in various plants such as *Arabidopsis*, tobacco, potato, tomato, rice, wheat, and banana [4, 5, 13, 14]. Targeting various genes by employing CRISPR/Cas9 is a more relaxed approach in comparison to the other known genome modification tools. Therefore, it is considered as a most promising tool for metabolic engineering.

**Fig. 1:**
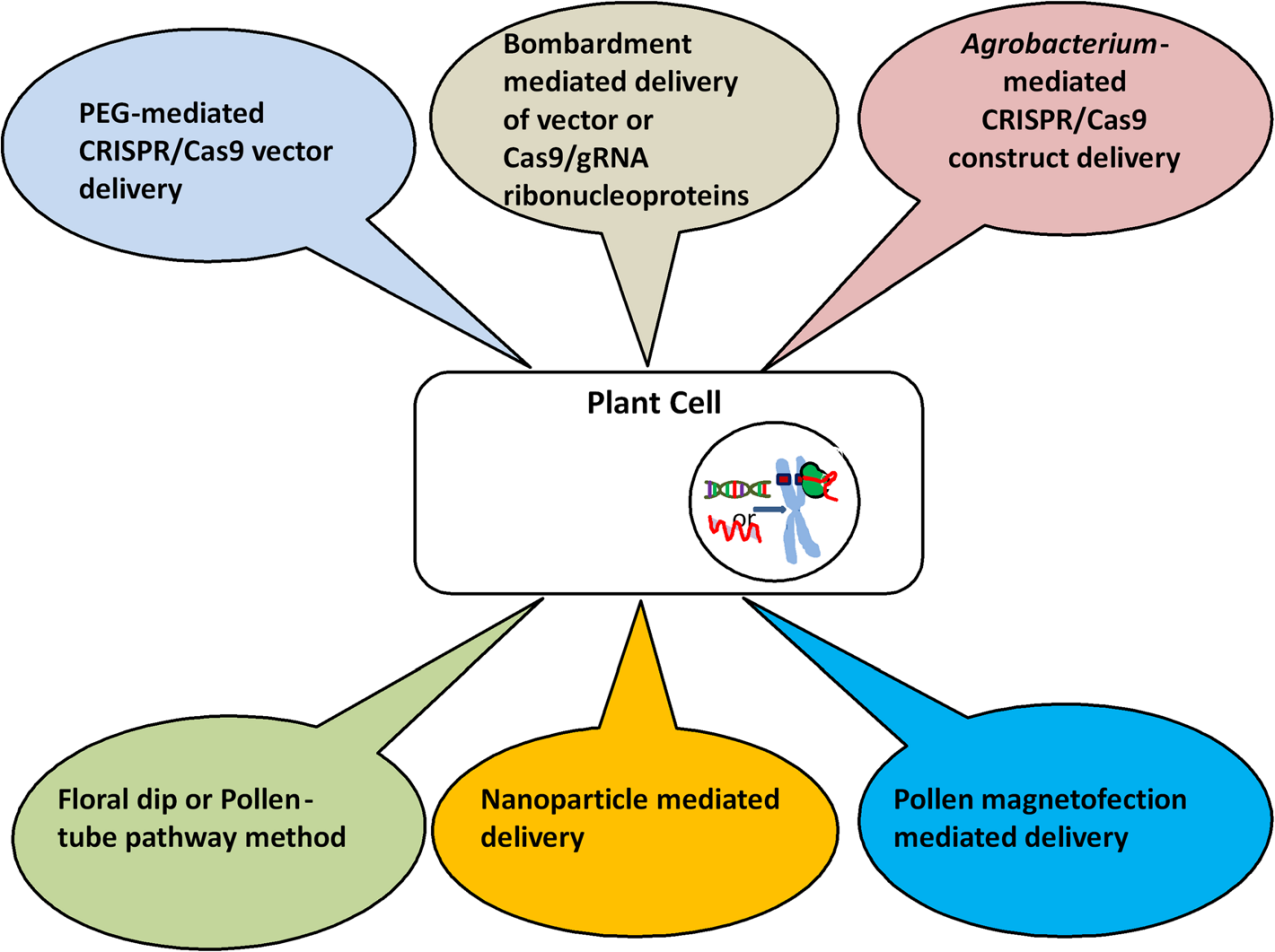
Existing and potential future CRISPR/Cas9 delivery methods. Different well-known delivery methods such as *Agrobacterium*-mediated delivery, Bombardment-mediated delivery, PEG-mediated delivery, and floral dip or pollen-tube tube pathway method. Potential pollen magnetofection-mediated delivery and nanoparticle-mediated delivery will be useful in near future to avoid tissue culture

The delivery of CRISPR/Cas9 components within rigid plant cells is a tough task. There are three methods of the construct delivery in plant cell: PEG mediated, *Agrobacterium*-mediated transformation, and bombardment or biolistic transformation. However, we insights the strength and weaknesses of each method of delivery depend upon plant species. We have elaborately discussed two potential methods for CRISPR/Cas9 vector-nanoparticle complex and a novel pollen magnetofection-mediated delivery in plants that would be most useful shortly (Fig. 1).

## Main text

### PEG-mediated CRISPR/Cas9 vector delivery

**Fig. 2:**
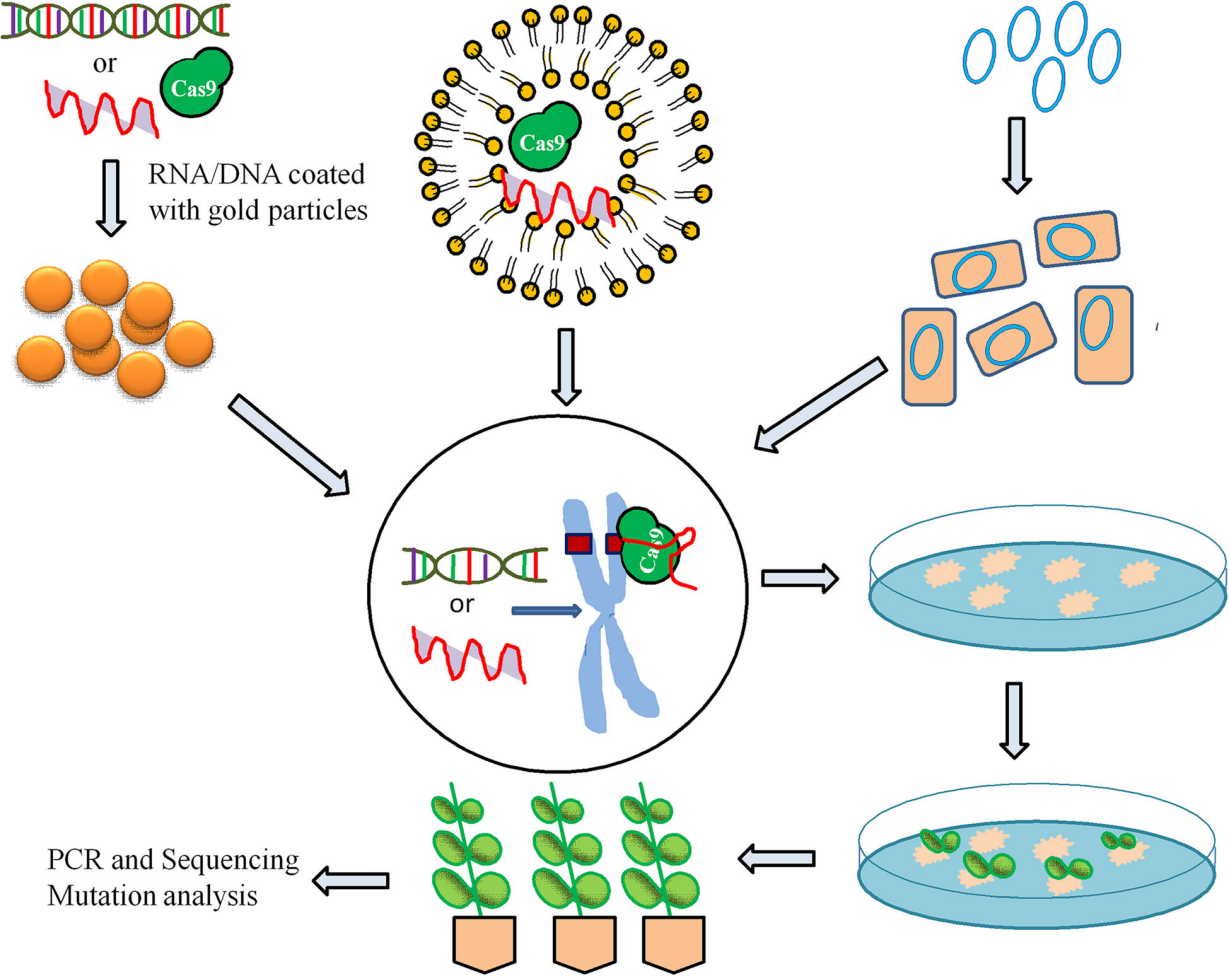
Schematic representation of CRISPR/Cas9 construct transfer and genome editing processes. CRISPR constructs coated onto gold particles (Gene gun mediated), transferred CRISPR construct into protoplast via PEG, and *Agrobacterium* strain having CRISPR vector. In the next step, CRISPR constructs are delivered into initial explants. A single plant cells and pictorial representation of gRNA and Cas9 at their target site within the genome. Further, the transformed explants are selected onto appropriate medium in plate. The survived plants were transferred into pots for acclimatization. PCR and Sanger sequencing are generally done for mutation screening

This is an important genetic transformation method carried out in the presence of polyethylene glycol (PEG) (Fig. 2). This method is successfully performed in protoplasts of different plants such as in maize, soybean, *A. thaliana*, tobacco, rice, and wheat, as summarized in Table 1. The plasmid-containing Cas9 and gRNA is incubated with protoplast in the presence of PEG. The PEG-mediated CRISPR construct delivery was firstly reported in maize and in this U3, and CaMV35S promoters were used for gRNA and Cas9, respectively [25]. In a few studies, Cas9 was expressed under some specific promoters, designed for specific plants, and to target crucial gene [27]. Plasmid DNA is dissolved in water and filtered to make it sterile and then mixed with protoplast suspension. After a few minutes, the required concentration of PEG is mixed slowly to protoplasts. After that, protoplasts are regenerated with a suitable regeneration medium [28]. Cas9/gRNA ribonucleoproteins were used to make transgene-free mutant plants in rice, *Arabidopsis*, tobacco, and lettuce using PEG-mediated delivery. The editing frequency in lettuce mutants was up to 46% [29]. Later, Kim et al. used Cpf1/CrRNA ribonucleoproteins to edit the genome of soybean and tobacco without using vector or DNA [30]. Cpf1 (CRISPR from *Prevotella* and *Francisella* 1) is an endonuclease of class II and type V CRISPR system. It identifies a thymidine rich PAM (TTTN) in a target location. Cpf1-mediated genome editing requires only crRNA, whereas Cas9 requires both crRNA and tracerRNA [30].

**Table 1:** PEG mediated CRISPR/Cas9 components delivery into different plants

Plant name	CRISPR/Ca9 vector or ribonucleoprotein complexes	Targeted genes	Reference
Apple	Cas9-sgRNA ribonucleoprotein complexes	*DIPM-1*, *2*, 4	[15]
*Brassica oleracea*, *Brassica rapa*	Cas9-sgRNA ribonucleoprotein complexes	*FRI*, *PDS*	[16]
*Citrullus lanatus*	PHSN1, PHSN2	*ClPDS*	[17]
*Glycine max*	pCas9-GmU6-sgRNA, pCas9-AtU6-sgRNA	*Glyma08g02290*, *Glyma12g37050*, *Glyma06g14180*	[18]
Grapevine	Cas9-sgRNA ribonucleoprotein complexes	*MLO-7*	[15]
*Oryza sativum*	pRGE3, pRGE6	*OsMPK5*	[19]
*Oryza sativum*	pUC19-OsCas9	*OsSWEET14*, *OsSWEET11*	[20]
*Oryza sativum*	pJIT163-2NLSCas9	*OsPDS*, *OsBADH2*	[21]
*Petunia*	Cas9-sgRNA ribonuclease protein complexes (RNPs)	*PhACO1*	[22]
*Physcomitrella patens*	pAct-Cas9, psgRNA PpAPT-KO4, PpAPT-KO7	*PpAPT*	[23]
*Solanum tuberosum*	Cas9-sgRNA Ribonucleoprotein complexes (RNPs)	*GBSS (GT4)*	[4]
*Triticum aestivum*	pCR8-U6-gRNA	*TaEPSPS*	[24]
*Zea mays*	p ZmU3-gRNA	*ZmIPK*	[25]
*Zea mays*	CT-nCas9	*ZmALS1*, *ZmALS2*	[26]

The main strength of the PEG-mediated delivery method is widely used to deliver Cas9/gRNA ribonucleoproteins. The vector less or DNA-free edited mutants of plants will be more acceptable and probably no issue with regulatory and ethical barriers. In 2016, transgene-free editing of six *polyphenol oxidase* mushroom was generated using ribonucleoproteins and PEG mediated. The mutant mushroom showed a 30% reduced enzyme activity responsible for browning, and it also escapes US regulation [31]. This ribonucleoprotein complexes cannot be delivered by widely used *Agrobacterium* mediated or floral-dip method.

The significant challenges and weakness of PEG-mediated delivery method is the establishment of suspension cells and protoplasts isolation. Apart from this, the main hurdle of regeneration of protoplasts into whole plants is in the case of recalcitrant plants. Due to this reason, the alternate methods of delivery of Cas9/gRNA ribonucleoproteins require to explore for efficient genome editing.

### Bombardment-mediated delivery of vector or Cas9/gRNA ribonucleoproteins

This method of transformation or gene transfer needs a device known as “gene gun or biolistic gun”. Generally, gold, silver, and tungsten particles are used as the carrier for vectors or Cas9/gRNA ribonucleoproteins (Fig. 2). CRISPR/Cas9 components are transferred through coated particles into explants by applying high pressure. This method requires optimized conditions such as helium pressure, target distance, particle size, and type of explants used. The transformed explants are regenerated onto regeneration medium with appropriate selection pressure. The successful delivery of Cas9/gRNA ribonucleoproteins and successive regeneration of mutants have been reported in maize [32], potato [4], and brassica [16], as tabulated in Table 2. Due to vector/DNA less editing, the Cas9/gRNA ribonucleoprotein delivery by the bombardment method is in demand. The regeneration of transformed tissues and selection pressure is tedious, and therefore, very low editing efficiency is achieved in maize 2.4 to 9.7% [32]. Recently, Cas9 with cytidine base editor along gRNA was in vitro transcribed and used for delivery into protoplast of wheat and rice for base editing [39].

**Table 2:** Particle bombardment method for CRISPR/Cas9 component delivery

Plant name	CRISPR/Cas9 vector or RNP complex	Selectable marker	Target genes	Reference
*Glycine max*	QC810 and RTW830*,* QC799 and RTW831	*HptII*	*DD20*, *DD43*	[33]
*Hordeum vulgare*	pcas9:sgRNA	*HptII*	ENGase	[34]
*Oryza sativum*	pCam1300-CRISPR-B	*HptII*	*crtI*, *ZmPsy*	[35]
*Oryza sativum*	CRISPR-RNP complex	*HptII*	*OsPDS1*	[36]
*Oryza sativum*	pJIT163-2NLSCas9	*HptII*	*OsPDS, OsBADH2*	[21]
*Oryza sativum*	pOsU3-sgRNA, pJIT163-2NLSCas9	*HptII*	*OsPDS*, *OsDEP1*	[37]
*Triticum aesituvam*	pJIT163-Ubi	*bar*	*TaMLO-A1*, *TaMLO-B1*, *TaMLO-D1*	[38]
*Zea mays*	pSB11-Ubi:Cas9	*Pat*	*LIG1*, *Ms26*, *Ms45*, *ALS1*, *ALS2*	[32]

The main strength of this delivery method is there is no necessity of the CRISPR/Cas9 binary vector. Several types of explants can be transformed with large and multiple DNA and RNA can be delivered. The most important is the Cas9/gRNA ribonucleoprotein complex which can also be delivered efficiently. The main drawback of this delivery method is random integration patterns within the genome, relatively less editing efficiency, costlier than others, and the bombardment sites such as cytoplasm, nucleus, mitochondria, or plastid cannot be controlled [40].

### *Agrobacterium*-mediated CRISPR/Cas9 construct delivery

Out of all delivery methods, this is the most widely used for a wide range of plant species. The binary vector that contains Cas9 and the gRNA expression cassette is transformed into the *Agrobacterium* strain. Further, the *Agrobacterium*-mediated genetic transformation of CRISPR constructs into the desired explant such as callus, leaf, and floral organs of plants (Fig. 2). To date, more than 20 plant species were efficiently edited with the help of *Agrobacterium*-mediated delivery of CRISPR/Cas9 components, summarized in Table 3. *Agrobacterium*-mediated transformation is more efficient and showed high editing efficiency than the particle bombardment method. Monocot crops that have less regeneration and transformation capacity are also frequently used for genetic transformation utilizing *Agrobacterium*. The CRISPR/Cas9 binary vectors suitable for *Agrobacterium* for high editing efficiency for monocot and dicot were designed [61]. *Agrobacterium*-mediated genome editing of banana cultivar Rasthali showed 59% mutation frequency of the *phytoene desaturase* gene [13]. In another report, 100% editing efficiency was reported in Cavendish banana cultivar “Williams” using *Agrobacterium*-mediated editing of the same gene [62]. Out of all delivery methods, *Agrobacterium* mediated is the most promising and useful method even for woody plants. The woody plants such as *Citrus sinensis* and poplar were efficiently edited by *Agrobacterium*-mediated genome editing [63, 64]. These methods are generally useful for the plant that is easily transformed through the leaf, callus, and floral organs.

**Table 3:** *Agrobacterium*-mediated delivery of CRISPR/Cas9 components in different plant species

Plant name	CRISPR/Cas9 vector	Selectable marker	Strain	Target genes	Reference
*Arabidopsis thaliana*	pUC119-RCS	Marker free	GV3101	*AtPDS3*, *AtFLS2*, *RACK1b*, *RACK1c*	[41]
*Arabidopsis thaliana*	pCAMBIA1300	*HptII*	GV3101	*BRI1*, *GAI*, *JAZ1*	[42]
Banana	pRGEB31	*HptII*	AGL1	*RAS-PDS*	[13]
Banana	pRGEB31	*HptII*	AGL1	*LCYε*	[43]
*Citrus sinensis*	pCas9-GN	*NptII*	LBA4404	*CsWRKY22*	[44]
*Cucumis sativum*	pRCS	*NptII*	EHA105	*eIF4E*, *eIF(iso)4E*	[45]
*Glycine max*	p201N Cas9	*NptII*	K599	*GFP transgene*	[46]
*Kiwi fruit*	pHLW-sgRNA-Cas9-AtU6-1, pPTG-sgRNA-Cas9-U6-1	*NptII*	EHA105	*AcPDS*	[47]
*Lotus japonicus*	pCAMBIA1300	*HptII*	EHA105	*LjLb1*, *LjLb2*, *LjLb3*, *LjSYMRK*	[48]
*Marchantia polymorpha*	pMpGE013 and pMpGE014	*HptII*	*–*	*MpARF1*	[49]
*Medicago trancatula*	pMDC32-AtU6-26	*HptII*	ARqual	*GUS*	[50]
*Medicago truncatula*	pFGC5941	*Bar*	*–*	*MtPDS*	[51]
*Nicotiana benthamaina*	pICH86966	*–*	AGL1	*NbPDS*, *PDS*	[14]
*Nicotiana benthamaina*	pUC19, pKQ334	*HptII*	GV3101	*NbPDS3*, *NbIspH*	[52]
*Nicotiana tabaccum*	pORE	*NptII*	LBA4404	*NtPDS*, *NtPDR6*	[53]
*Oryza sativum*	VK005	*HptII*	EHA105	*ISA1*	[54]
*Populous tomentosa*	pYLCRIPSR/Cas9, pUC18	*HptII*	*–*	*PtoPDS*	[55]
*Salvia miltiorrhiza*	pCAMBIA1300	*HptII*	C58C1	*SmRAS*	[56]
*Solanum lycopersicum*	pYLCRISPR/Cas9	*HptII*	*–*	*SGR1*, *LCY-E*, *Blc*, *LCY-B1*, *LCY-B2*	[57]
*Solanum lycopersicum*	pENTR-sgRNA: pMR290/Cas9	*NptII*	EHA105	*SlCCD8*	[58]
*Solanum tuberosum*	pMDC32	*HptII*	*–*	*StALS1*	[59]
*Sorghum bicolor*	pVS1 binary vector derived from pLH7500	*NptII*	Y158	*DsRED2*	[20]
*Triticum aesituvam*	pBI121	*NptII*	GV3101	*Inox*, *PDS*	[14]
*Zea mays*	pMCG1005	*Bar*	EHA101	*Argonaute 18*, *Dihydroflavonol-4-reductase*	[60]

The significant advantage of this method is high editing efficiency compared to other known methods. Another advantage of this method is broad applicability, easily available, and less costly than others. By this method, stable transgene integration is achieved, mostly with single-copy integration [65]. Due to all these properties, to date, it is a widely accepted method of amongst all known methods. The only drawback of this method is, it requires a binary vector, and it incorporates an alien gene into the plant genome.

### Floral-dip or pollen-tube pathway method

The earlier plasmids was either directly applied onto the surface of stigma or mixed with pollen and then applied to their receptive stigma [66]. Further, various parameters were optimized for efficient gene transfer such as wounding and dipping of flowers having male and female organs into *Agrobacterium* suspension. For the successful floral transformation of plants, the stage of plants is crucial. Apart from various physical parameters such as media composition, pH, optical density, temperature, and humidity, molecular factors such as choice of the promoter, gene size, and vector types are also important. The well-known constitutive promoter such as CaMV35S and *Arabidopsis* UBI10 was also involved for increased editing efficiency. UBI10 promoter was found more efficient in the germline of *Arabidopsis* [5]. Further, the germline-specific promoter, such as MGE1, YAO, RPS5a, AG, and ICU2 promoters, was used for efficient editing [5]. The highest editing efficiency was obtained using RPS5a and YAO promoters [5]. Egg cell-specific promoter EC1.2 and EC1.2::EC1.1 has also exhibited substantial editing efficiency with CRISPR/Cas9 in *Arabidopsis* [67]. To date, the floral dip-mediated genome editing is limited to only *Arabidopsis* [5, 67]. However, the successful plant genetic transformation was done in flax, tomato, radish, *Brassica rapa,* and wheat, *Setaria viridis* using floral dip [68–71]. The 50–60% transformation efficiency was reported in flax, which is higher than those reported for *Arabidopsis* using the floral-dip method of gene transfer [69].

The main advantage of this delivery method did not require a plant tissue culture facility. Floral-dip-mediated delivery of CRISPR/Cas9 components is cost-effective and straightforward. This method is most widely and commonly used for *Arabidopsis* genome editing across the world. The drawback of floral-dip-mediated delivery of CRISPR/Cas9 components is limited to few plants such as *Arabidopsis,* flax, and tomato etc, with less efficiency due to limited flower and seed formation.

### Knock-in through the sequential floral-dip method

Site-directed insertion of the desired gene or promoter or desired segment of DNA at a specific location by CRISPR/Cas9 is in demand. This has been successfully demonstrated in tomato, maize, wheat, and potato. In this, a donor template or donor vector is required, which consists of the left and right homology arm. For example, one of the donor vectors consists of two T-MLO homology arms and a GFP coding sequence. This constructed GFP donor vector transferred into wheat protoplast for GFP knock-in along with the CRISPR/Cas9 vector [38]. CRISPR/Cas9 component, along with the donor vector, was used in soybean callus. This donor DNA is consists of soybean specific promoter and *HptII* gene that confer hygromycin resistance [33]. The knock-in using CRISPR can be done into the germline cells or other regenerative cells with the help of the donor vector. The *Arabidopsis* line carrying Cas9 was used for sequential floral-dip method of transformation using germline-specific promoters such as DD45, Lat52, YAO, and CDC45 promoter [72]. The Cas9 regulated with DD45 promoter was found more efficient for knock-in, with the high rate of editing in egg cells or early embryos as compared to other regenerative tissues [72]. The floral-dip method of gene transfer has been demonstrated in various crops such as wheat [68], flax [69], radish [70], and tomato [71]. The Cas9, driven by egg cell or embryo-specific promoters along with the desired donor DNA template, might lead to efficient knock-in of targeted genes in different crops.

### Nanoparticle-mediated delivery

**Fig. 3:**
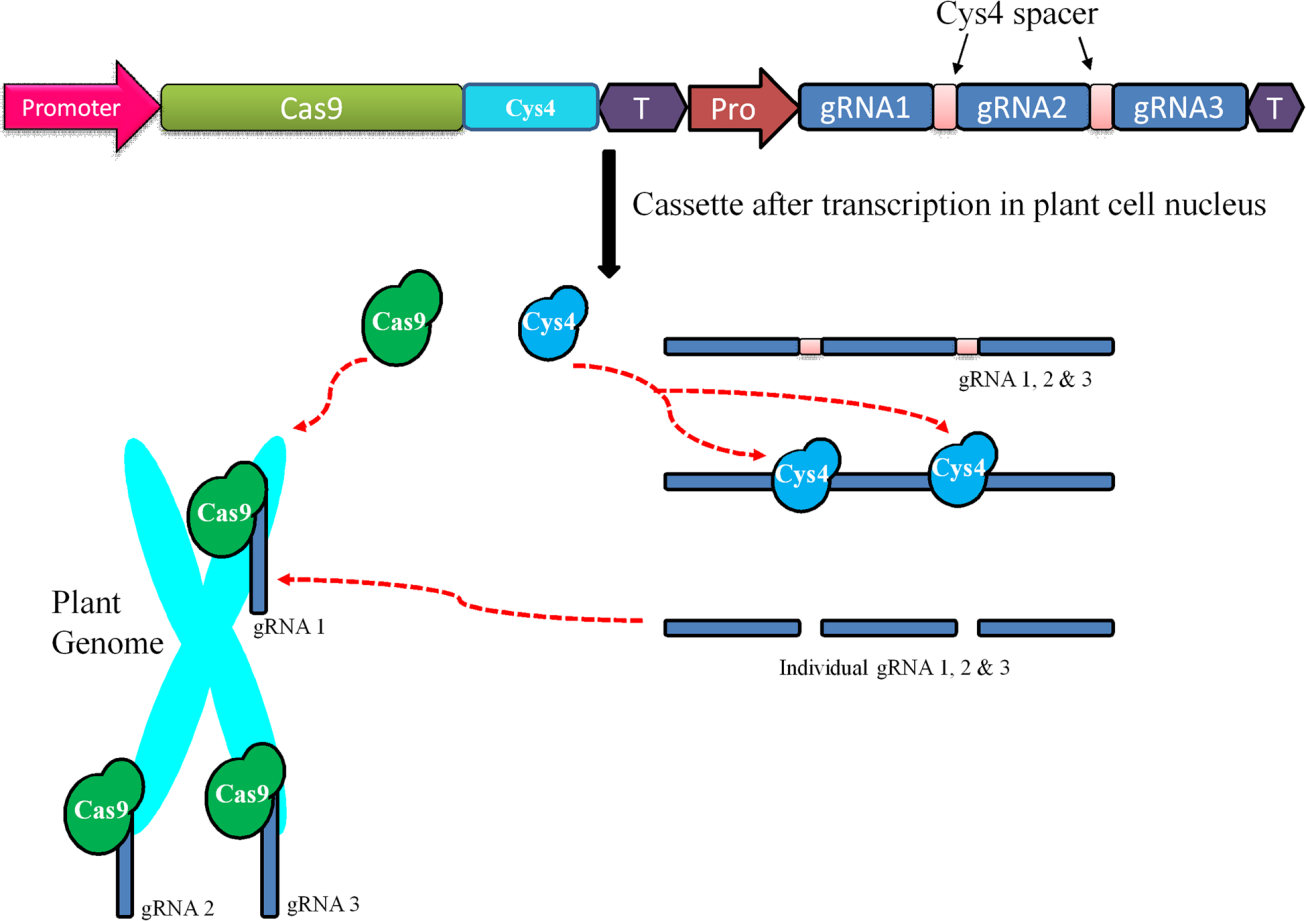
Mechanism of Cas9/polycistronic-Csy4-gRNA complexes mediated editing of the genome. A CRISPR/Cas9 construct containing promoter (Pro), *Cas9*, *Csy4*, terminator (T), and guide RNA (gRNA1, 2, and 3). Construct delivered into plant cells and transcribed into nucleus. The transcript of Cas9 and Cys4 will translated into proteins (nucleases), whereas multicistronic gRNA are separated into individual sgRNAs (dark blue boxes) due to action of Cys4 nucleases (sky blue circles). Further, gRNA1, 2, and 3 scan their respective target sites where Cas9 (green circles) binds and breaks the genome

To date, various efforts have been made to uptake different nanoparticles into dicot and monocot plant cells. The direct uptake of numerous nanoparticles includes mesoporous silica nanoparticles [73], carbon nanotubes [74], quantum dots [75], and metal/metal oxide NPs [76, 77]. The success of silicon carbide whisker-mediated genetic transformation of various crops such as maize [78], cotton [79], and rice [80] suggests that CRISPR/Cas9-mediated genome editing will be useful to generate transgene-free plants. Cas9/gRNA ribonucleoproteins, along with appropriate nanoparticles, can be delivered into regenerative tissues. The multiple gRNAs, along with appropriate promoters and terminators, into a single plant transformation vector, are required for modulating multiple pathways (Fig. 3). However, the large size of a construct or multiple gRNAs will be tedious for delivering into plant cells. Therefore, polycistronic-tRNA-gRNA or polycistronic-Csy4-gRNA, along with nanoparticles, will be effective for multiple editing via non-transgenic approaches. The success of every delivery method depends upon the method used as well as successive regeneration into whole plants. The plant protoplast is the primary target for the delivery of CRISPR/Cas9 components. However, less regeneration frequency leads to lower editing efficiency.

The main advantage of nanoparticle-mediated delivery of CRISPR/Cas9 components is not limited to a protoplast. We can directly deliver it into plant regenerative tissues. The disadvantage of this method is less efficient and needs suitable nanoparticles with high carrying capacity CRISPR/Cas9 components.

### Pollen magnetofection-mediated delivery

Magnetofection is a technique of genetic transformation that utilizes the magnetic force for the uptake of vector allied with magnetic nanoparticles (MNP). In this method, positively charged polyethyleneimine-coated Fe_3_O_4_ MNPs and negatively charged vector are used to form MNP-DNA complexes. Further, the pollens are mixed with the complexes, and a magnetic field is applied. Then, the pollens were applied for pollination. This technology has successfully applied to cotton [81]. Nowadays, two methods are frequently applied for delivery of CRISPR/Cas9 components: the first one is CRISPR/Cas9 vectors, and the second is vector/DNA less CRISPR/Cas9 system. The vector/DNA-free editing by magnetofection will be beneficial for generating nontransgenic crops. The following two methods are used to achieve this: (i) Transcribed sgRNA and Cas9 mRNA: The gRNA and Cas9 are transcribed in vitro and then coated with MNP and delivered to stigma or protoplast. For in vitro transcription, gRNA and Cas9 are regulated by a T7 promoter. The T7 RNA polymerase can be used for in vitro transcription and finally treated with DNase I. For example, in vitro mRNA transcripts of gRNA and Cas9 were co-bombarded into wheat calli [82]. (ii) Transcribed sgRNA and purified Cas9 protein: Cas9 protein and transcribed sgRNA can be attached to the MNP and then mix with pollens. Further, these can be transferred onto the stigma for fertilization or directly regenerated by tissue culture for mutant haploid production. Vector/DNA-free genome editing was shown in *Arabidopsis*, tobacco, lettuce, and rice [29]. Fruit crops such as grapevine and apple were also edited by the vector-free method using CRISPR/Cas9 ribonucleoproteins [83]. In maize, these complexes were bombarded in the embryo, and transgene-free mutant was recovered [32].

To date, there was no report for pollen magnetofection-mediated genome editing. However, the advantage of this method is we can directly transfer the CRISPR/Cas9 ribonucleoproteins into pollens. This can save the time required for tissue culture and selection of transgenic.

### Factors affecting editing efficiency

Different factors such as selectable marker, promoter regulating *Cas9* gene and gRNA, *Agrobacterium* strain, PEG concentration, temperature, and bombardment pressures might affect mutation frequency. Higher expression of the *Cas9* gene and gRNA depends on the correct promoter, and finally, it leads to higher mutation. Recent studies showed that U6 small nuclear promoter (U6) and human H1 Pol III promoter (H1) promoters efficiently express gRNA. H1, U6, and U3 promoters are RNA polymerase III promoters that efficiently transcribe short non-coding transcripts. Therefore, these promoters are used to regulate 75 bp short gRNA. However, the H1 promoter is more useful than U6 because transcription can be initiated with any nucleotide in the case of H1 promoter, whereas U6 requires a G nucleotide. The robust H1 polymerase III promoter efficiently regulates gRNA [84]. T7, T3, and SP6 promoters are RNA polymerase promoters characterized from bacteriophage. These promoters require initiating G nucleotide and used in in vitro transcription of gRNA and Cas9 [84]. The strength of the promoter estimated by comparing the percentage of mutation which occurs in the plant. In *Zea mays*, the ubiquitin promoter showed more efficiency than the CaMV35S [85]. Ubiquitin (Ubi) and Cauliflower mosaic virus (CaMV35S) promoters are well known as a constitutive promoter, and it expresses every tissue of plants. DNA-dependent RNA polymerase III (pol III)/U3 promoter and pol III terminator are used to drive the gRNA expression. pRGE3 and pRGE6 vectors use *Oryza sativum* pol III (SnoRNA U6 and U3) promoter. The *Arabidopsis* AtU6 and AtU3 promoters are frequently used to regulate gRNA in dicots plants, whereas OsU6 and OsU3 are used in monocot plants [12, 28, 86].

Cas9 from *Streptococcus pyogenes* (SpCas9)was more active at 37 °C as compared to 22 °C during the in vitro assay. SpCas9 was used for genome editing in *Arabidopsis*. It showed higher editing efficiency when *Arabidopsis* were subjected to heat stress at 37 °C as compared to 22 °C [87]. Genome editing using Cpf1 requires higher temperatures for efficient editing efficiency. The *Lachnospiraceae bacterium* Cpf1 showed less editing efficiency at 22 °C, whereas it showed 100% editing efficiency at 28 °C [88]. CRISPR/Cas9-mediated genome editing of citrus also showed higher editing efficiency when plants were subjected to heat stress at 37 °C [87]. These all results suggest that temperature is also an essential factor affecting the mutation frequency.

### Advancement in CRISPR technology and future perspective

Recent advancements in CRISPR technology are CRISPR interference (CRISPRi) and CRISPR mediated activation (CRISPRa). Different types of vectors for this are available commercially to open up new ways in genome engineering. CRISPRi and CRISPRa had been used in plants to control gene expression at the transcriptional level [89, 90]. The deadCas9 (dCas9) catalytically inactive is used in these techniques. The dCas9 alone or fused with a transcriptional repressor is used in CRISPRi technology. This chimeric dCas9 protein binds at their respective target site within the promoter region. Due to this, RNA polymerase activity is suppressed, and finally, the transcription is blocked [7, 89, 90]. In CRISPRa, dCas9 fused with transcriptional activators and therefore enhanced transcription as compared to normal promoter strength [28]. Cas9 nickases are produced by mutating RuvC (D10A) and HNH (H840A) domains of wild-type Cas9 [91]. It can cleave only one strand of DNA and therefore used as a paired to break both strands. Paired Cas9 nickases have less off-target activity as compared to wild-type Cas9 plants such as *Arabidopsis* and rice [91]. Cas9 nickases are useful for targeted gene insertion in plants.

All these chimeric proteins and variant Cas9 proteins need to be delivered into plant cells efficiently. To date, this has been achieved by *Agrobacterium*-mediated genetic transformation. *Agrobacterium*-mediated transformation of rice, tobacco, and *Arabidopsis* was done for delivery CRISPRi and CRISPRa construct [28, 61]. The delivery of this chimeric Cas9 protein along with multiple gRNA without using vector will be useful for crop improvement. However, the delivery of in vitro transcribed polycistronic tRNA-gRNA or Cys4-gRNA unit along with purified Cas9 would be a challenge. Probably, pollen magnetofection mediated and nanoparticle-mediated delivery will solve these issues and generate non-transgenic mutant plants.

## Conclusion

The high efficiency of genome editing depends upon various factors. However, the most important is the type of delivery method used. In banana, 100% editing efficiency was reported using *Agrobacterium*-mediated delivery of CRISPR/Cas9 components [62]. The success of every delivery method depends upon the tissue type and successive regeneration into whole plants. These concerns of regeneration depend upon the nature of plant species, tissue type, and culture method. Therefore, this publication emphasized the need to develop new methods for delivery of CRISPR/Cas9 components such as nanoparticle-mediated delivery and pollen magnetofection mediated delivery. These two potential methods of delivery into pollen or directly into the meristematic region would allow researchers to omit the time consuming and laborious tissue culture. The authors expected these novel delivery methods would boost up the CRISPR/Cas technologies in agriculture. The crops with altered genome will be cross the barrier of ethical as well as regulatory issues because it does not require any vector of DNA for editing.

## Data Availability

Not applicable for this article.

## Abbreviations

gRNA: Guide RNA
RGNs: Site-specific nucleases
PAM: Protospacer Adjacent Motif
DSBs: Double-stranded breaks
HDR: Homologous direct repair
NHEJ: Non-homologous end joining
PEG: Polyethylene glycol
MNP: Magnetic nanoparticles
